# On the Encoding of Proteins for Disordered Regions Prediction

**DOI:** 10.1371/journal.pone.0082252

**Published:** 2013-12-16

**Authors:** Julien Becker, Francis Maes, Louis Wehenkel

**Affiliations:** 1 Bioinformatics and Modeling, GIGA-Research, University of Liege, Liege, Belgium; 2 Department of Electrical Engineering and Computer Science, Montefiore Institute, University of Liege, Liege, Belgium; 3 Declaratieve Talen en Artificiele Intelligentie, Departement Computerwetenschappen, University of Leuven, Leuven, Belgium; Universita' di Padova, Italy

## Abstract

Disordered regions, *i.e.*, regions of proteins that do not adopt a stable three-dimensional structure, have been shown to play various and critical roles in many biological processes. Predicting and understanding their formation is therefore a key sub-problem of protein structure and function inference. A wide range of machine learning approaches have been developed to automatically predict disordered regions of proteins. One key factor of the success of these methods is the way in which protein information is encoded into features. Recently, we have proposed a systematic methodology to study the relevance of various feature encodings in the context of disulfide connectivity pattern prediction. In the present paper, we adapt this methodology to the problem of predicting disordered regions and assess it on proteins from the 10th CASP competition, as well as on a very large subset of proteins extracted from PDB. Our results, obtained with ensembles of extremely randomized trees, highlight a novel feature function encoding the proximity of residues according to their accessibility to the solvent, which is playing the second most important role in the prediction of disordered regions, just after evolutionary information. Furthermore, even though our approach treats each residue independently, our results are very competitive in terms of accuracy with respect to the state-of-the-art. A web-application is available at http://m24.giga.ulg.ac.be:81/x3Disorder.

## Introduction

Disordered regions refer to regions in proteins that do not adopt a stable three-dimensional structure when they are not in presence of their partner molecules. Over the last decade, several experimental studies have shown that proteins with disordered regions play various and critical functions in many biological processes. The flexibility of these regions makes it possible for a protein to interact, recognize and bind to many partners. For example, disordered regions are often involved in regulatory and signaling interactions [2] such as the regulation of cell division, the transcription of DNA or the translation of ARNm. They also play a role in the self-assembly of protein complexes, and in the storage of small molecules [3], [4].

Several automatic methodologies have been proposed to predict disordered regions from primary sequences. They range from simple methods based on the sequence complexity [5] to more sophisticated machine learning approaches often relying on neural networks or Support Vector Machines (SVMs)[6]–[10]. For example, the Poodle tool is based on three adjacent classifiers, which are specialized in making short [11] or long [12] disordered regions predictions, or unfolded protein predictions [13], while the Spritz tool [14] uses two specialized SVMs for either short or long disordered regions. Recently, meta-predictors have also appeared in the literature. These approaches consist in combining predictions of a large number of existing disordered regions predictors [15], [16], *e.g.*, GSmetaDisorder gathers no less than 12 different predictors. Nowadays, there exist more than 50 disordered region predictors. Fortunately, since 2004, a part of the biannual competition “*Critical Assessment of Techniques for Protein Structure Prediction*” (CASP) is devoted to the comparison of the participant disordered regions predictors. For more information about disordered regions predictors, one can refer to the reports of these assessments [17] or to the recent comprehensive overview of computational protein disorder prediction methods made by Deng *et al.* [18].

In machine learning, the way to encode information into vectors of features typically has a major impact on the classification accuracy. In the context of bioinformactics, and specifically in the case of protein structure inference, candidate features are typically grouped into parameterized families of features (we use the term ‘feature function’ to denote such a family), where each family provides a different kind of physical or biological information. Recently, we have developed a systematic feature function selection methodology [1] for the inference of disulfide bridges within protein structures, and which allowed us to identify a minimal subset of relevant feature functions for this problem.

The main contribution of the present paper is the adaptation of the selection pipeline presented in our previous work [1] to establish a relevant representation of residues in the context of disordered regions prediction. For this purpose, we consider various feature encodings and, in addition to the primary structure, three in-sillico annotations: position-specific scoring matrices (PSSM), predicted secondary structures and predicted solvent accessibilities. We apply the feature function selection pipeline in combination with Extremely randomized Trees (ETs), a model which gave excellent results in previous work [1]. In order to avoid any risk of overfitting or over-estimation of our models, we use three distinct datasets: Disorder723 [19], Casp10 (http://www.predictioncenter.org/casp10/) and Pdb30. We first apply feature selection on Disorder723 and then assess the relevance of the selected feature functions both on Casp10 and on Pdb30.

The main result of our study is to highlight a novel feature function encoding the proximity along the primary sequence of residues predicted as being accessible (resp. inaccessible) to the solvent. This feature function is identified as the second most important for predicting the belonging of a residue to a disordered region, just after evolutionary information derived from the PSSM. To our best knowledge, these features encoding solvent accessibility have never been highlighted in previous studies of disordered regions prediction. The majority of the remaining relevant feature functions that we found (e.g., evolutionary information and sequence complexity) were already suggested by other studies of disordered regions [5], and we thus confirm in a fair way their relevance. Furthermore, even though our approach treats each residue independently, *i.e.*, without explicitly modelling global properties of disordered regions, our predictors are very competitive in terms of accuracy with respect to Casp10 assessments and to our very large independent test set extracted from Pdb30.

## Materials and Methods

There exist a huge number of manners to encode proteins into an appropriate form for machine learning algorithms, *i.e.*, vectors of (categorical or numerical) features. In this study, we consider a number of *feature functions*, which aim at encoding a particular property of the protein into a vector of features of fixed length. For example, the enumeration of the 11 amino acids at the flanks of a residue of interest is a feature function that, given a residue position within a protein, returns a vector of 11 categorical features. To form more sophisticated representations, feature functions can be combined through the concatenation of their encoding vectors.

Among the large number of possible combinations, our study aims at identifying the minimal feature function set that is relevant for disordered regions prediction. In [1], this identification is performed through a forward feature function selection algorithm for the problem of disulfide bridge prediction. In order to work, this algorithm requires four components to be specified: a dataset, a list of candidate feature functions, a base learner and a criterion to optimize.

This section describes how we have adapted each of these four components to the problem of predicting disordered regions of proteins. The first part presents the three datasets (Disorder723, Casp10 and Pdb30) and how we enrich the primary structures of each of these datasets with three annotations: position-specific scoring matrices (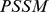), predicted secondary structures (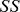) and predicted solvent accessibilities (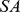). The second part of this section formulates disordered regions prediction as a supervised-learning problem and, more specifically, as a binary classification problem, which aims at predicting the disorder state (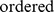 or 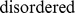) of each protein residue. It also defines five measures to assess the quality of the predictions. The third part briefly describes the forward feature functions selection methodology and enumerates the candidate feature functions that we consider during the selection process. Finally, the last part of this section introduces ensembles of extremely randomized trees, which are used as the base learner within the feature function selection algorithm.

### Datasets and annotations

This study relies on three datasets. The first one, Disorder723 (http://casp.rnet.missouri.edu/download/disorder.dataset), has been built by Cheng *et al.* [19] and was extracted from the Protein Data Bank [20] in May 2004. The dataset is made of 723 non-redundant chains that contain at least 30 amino acids in length and that were solved by X-ray diffraction with a resolution of around 2.5 Å. In order to reduce the over-representation of particular protein families, the dataset has been filtered by UniqueProt [21], a protein redundancy reduction tool based on the HSSP distance [22], with a cut-off distance of 10.

The second dataset, Casp10, is the one used during the 10th CASP competitions that took place in 2012. During the competition, the candidate predictors have to make blind predictions, *i.e*, they have to predict disordered regions of proteins close to being solved or close to being published and that have no detectable similarity to available structures. At the end, the candidate predictors were assessed on 94 experimentally determined proteins available for download on the official CASP website(http://predictioncenter.org/download_area/CASP10/targets/casp10.DR_targets.tgz). Note that unlike Disorder723, the way to resolve protein structures is not restricted to X-ray diffraction and that CASP10 also contains protein structures determined by NMR.

**Figure 1:**
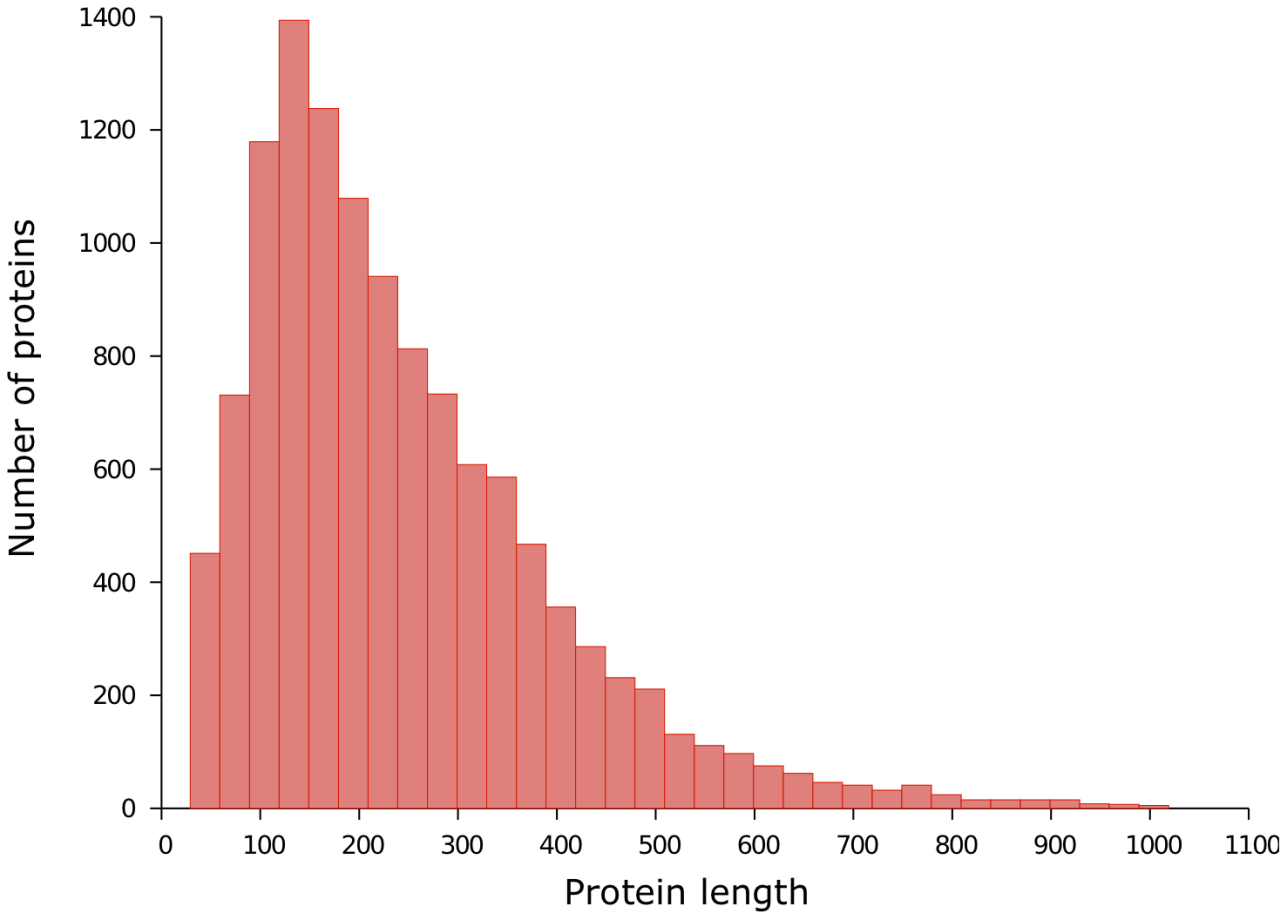
Protein length distribution of PDB30. There are 12,090 proteins. The average protein length is of 247.4 residues.

**Figure 2:**
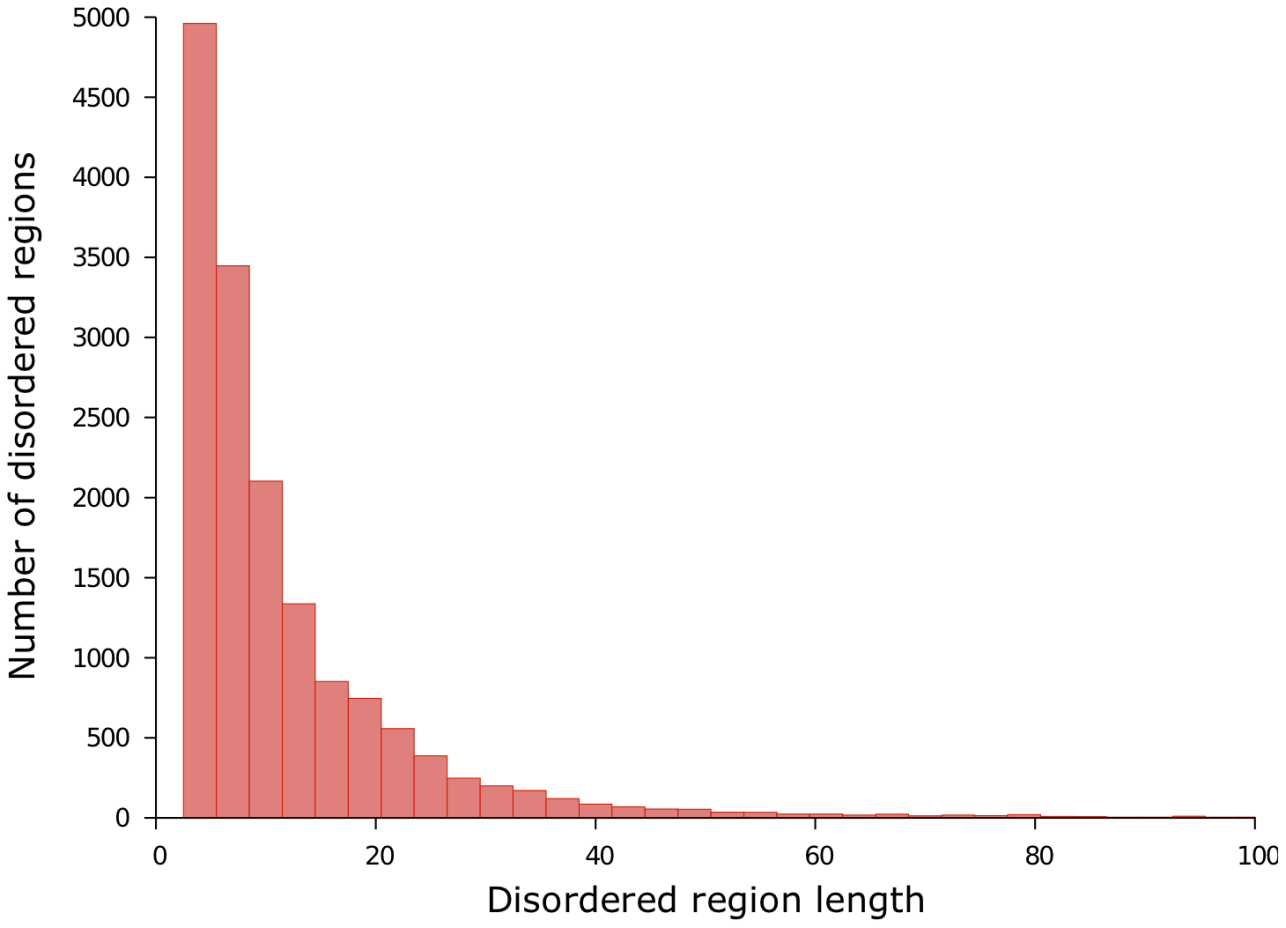
Disordered region length distribution of PDB30. There are 15,726 disordered regions. The average length of a disordered region is of 12.3 residues and the average number of disordered regions per protein is of 1.3.

The last dataset, that we denote by Pdb30, is far larger than the two previous ones. We created Pdb30 on one of the clustered versions of the Protein Data Bank (as of August 31, 2013) available at http://www.rcsb.org/pdb/statistics/clusterStatistics.do. The clustering is defined on a protein chain basis with a maximum pairwise sequence identity of 30%. The authors of this clustered version of PDB used BLASTClust [23] to perform the clustering and selected the representative structure of each cluster according to their quality factor. We then filtered out any proteins that were less than 30 amino acids in length, that had no X-ray structure or that had resolution coarser than 2.5 Å. Next, we discarded the proteins that share a sequence identity of at least 30% with a protein of Disorder723 (our training set). The final dataset is made of 12,090 proteins and 2,991,008 residues of which 193,874 (6.5%) are disordered. Figure 1 shows a histogram of the protein lengths. The average (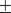 standard deviation) protein length is 247.4 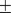 162.8. Figure 2 shows a histogram of the disordered region lengths of our dataset. The average disordered region length is 12.3 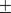 15.6. The dataset is available at: http://m24.giga.ulg.ac.be:81/x3Disorder/pdb30.dataset.

In our experiment, we use Disorder723 to identify a subset of relevant feature functions while Casp10 and Pdb30 are used to assess the quality of the selected feature functions. It is important to note that no protein in the Casp10 or Pdb30 sets share more than 30% sequence identity with one of those of Disorder723. This therefore makes it possible to fairly evaluate and compare our results with those that have participated to the 10th CASP competition.

We use the same definition of disorder as Cheng *et al.* and as the CASP competition, *i.e.*, segments longer than three residues but lacking atomic coordinates in the crystal structure are labelled as “disordered” whereas all other residues are labelled as “ordered”. According to this definition, Table 1 shows that the three datasets contain 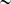 6% of disordered residues and 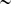 94% of ordered residues. Some residues in Casp10 were not classified by the CASP assessors. These residues were not taken into account in our experiments.

**Table 1:** Composition of datasets.

	Proteins	Ordered residues	Disordered residues	Residues
Disorder723	723	201,703 (93.55%)	13,909 (6.45%)	215,612
Casp10	94	22,688 (93.79%)	1502 (6.20%)	24,190
Pdb30	12,090	2,797,134 (93.52%)	193,874 (6.48%)	2,991,008

isorder723, Casp10 and Pdb30 datasets. All datasets have roughly the same proportion of disordered residues (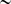 6%). Pdb30 contains 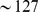 times more proteins and 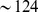 times more residues than Casp10. Number of proteins, number (and portion) of ordered/disordered residues and number of residues in D

We enrich the primary structure (denoted as 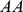) by using three additional annotations: evolutionary information in the form of a position-specific scoring matrix (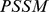), predicted secondary structure (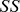) and predicted solvent accessibility (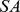). We computed the PSSMs by running three iterations of the PSI-BLAST program [24] on the non-redundant NCBI database [25]. To produce predicted annotations, we used the SSpro and ACCpro [3] programs for the predicted secondary structure (“helix”, “strand” or “coil”) and the predicted solvent accessibility (under or over 25% exposed), respectively.

### Problem statement

Let 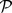 be the space of all proteins and 
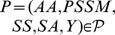
 one particular protein described as the 5-tuple containing its primary structure 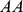, its 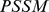, its two predicted annotations 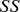 and 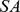, and its disordered regions 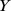. Each of these annotations is described as a sequence of 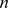 labels, where 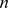 is the number of residues composing 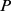. For example, the primary structure is defined as 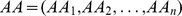, where 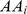 is the label corresponding to the amino acid of the 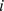-th residue of 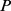, and the disordered regions annotation is defined as 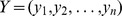, where 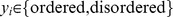. The disordered regions prediction task consists in assigning a label 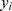 to each residue of 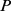.

In the supervised-learning formulation of the problem, we assume to have access to a dataset of proteins in which residues are labeled either 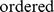 or 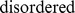. We denote this dataset 
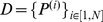
, where 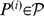 is the 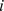-th protein. Given such a dataset 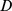, the aim is to learn a disordered regions predictor 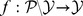 that maps a protein 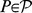 to a sequence 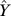 of 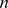 predicted labels 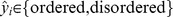, where 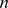 is the length of 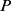.

It is important to note that disordered regions are segments, *i.e.* consecutive residues tend to share the same label. More and more machine learning approaches such as conditional random fields [27], recursive neural networks [19], meta-predictors [28] or post-filtering steps [29] are able to exploit the structured aspect of the problem.

However, as the goal of this study is to determine a set of relevant feature functions in general, we do not focus on such advanced prediction approaches here. We instead simplify the general problem into a standard binary classification problem. The aim is to learn a predictor 
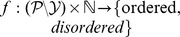
 that maps the 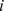-th residue of a protein 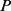 to the predicted label 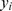. This formulation is rather simple in the sense that it treats each residue independently, *i.e.*, regardless with respect to predictions made on neighboring residues of the same protein.

#### Evaluation measures

In order to evaluate the quality of the predictions made by our models, we consider five residue-level performance measures: the balanced accuracy (Acc), the sensitivity, the specificity, the area under the ROC curve (AUC) and the F-measure. Each of these measures can be formulated using a tuple of four values: the number of *true positives* (TP), *false positives* (FP), *true negatives* (TN), and *false negatives* (FN), where a positive example is a disordered residue and a negative example is an ordered residue. Therefore, a true positive is a correctly predicted disordered residue and a false negative is an ordered residues falsely predicted as a disordered one.

According to these notations, the sensitivity [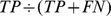] is the fraction of disordered residues that are successfully predicted as disordered, whereas the specificity [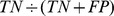] is the fraction of ordered residues that are successfully predicted as ordered. As the problem of disordered regions prediction is strongly imbalanced (only 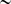 6% of residues are disordered), using the conventional accuracy may inflate performance estimate and is therefore not appropriate. However, the balanced accuracy, defined as the arithmetic mean of sensitivity and specificity, is robust against imbalanced datasets as well as the F-measure, which is used in recent CASP assessments. The F-measure is defined as the harmonic mean of the precision – the fraction of predicted disordered residues that are truly disordered – and the sensitivity (also called recall).

Since, a large number of available binary classifiers produce probabilities rather than strict classes, these criteria rely on a user-defined *decision threshold* to discriminate positive from negative examples. Depending on how users fixed their threshold, a bias might be introduced, which might lead to an unfair comparison between distinct studies. To tackle this issue, one can compare the performance of distinct models by their ROC curve, which is obtained by plotting the sensitivity against the false positive rate [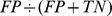] when varying the decision threshold. However, the comparison is not easy, especially when the curves are similar. A common simplification is therefore to calculate the area under the ROC curve (AUC). An area of 1.00 corresponds to a perfect predictor while an area of 0.50 corresponds to a random predictor.

### Forward feature function selection

**Figure 3:**
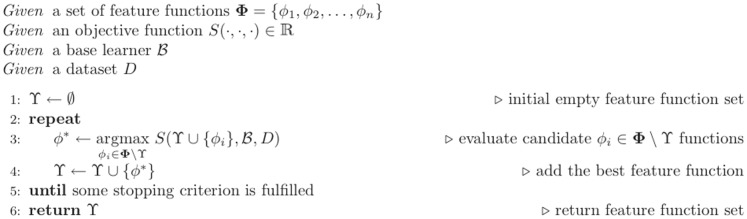
Forward feature function selection algorithm. In order to identify the relevant feature function set, the algorithm requires four components: a dataset, a list of candidate feature functions, a base learner and a criterion to optimize.

Recently, we have developed a tractable and interpretable feature function selection methodology [1], which aims at identifying a minimal set of relevant feature functions among a larger group of candidate feature functions. Note that this approach focuses on identifying feature functions rather than individual features. Figure 3 roughly depicts this algorithm. It is a *wrapper* approach that repeatedly evaluates subsets of feature functions through an objective function 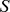, which typically cross-validates the base learner 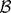 on a dataset 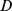, and that is directly driven by the scores returned by 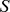. To obtain interpretable results, the method relies on a rather simple scheme, which consists in constructing the feature function set greedily in a forward way: starting from an empty set (line 1, in Figure 3) and adding (line 4) the feature function that maximizes 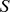 (line 3), to the current set of feature functions at each iteration. For a more detailed version of this algorithm, we refer the reader to our previous work [1].

The remaining of this section describes the list of our candidate feature functions. Some of these feature functions are identical to those presented in our previous work, while some others are a generalization of what we did previously and others are completely novel.

#### Candidate feature functions

The feature generation is performed through *residue feature functions* 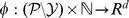 that, given the residue position 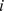 of a protein 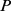, computes a vector of 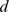 real-valued features.

Among the panel of candidate functions 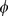 already described in our previous work, we adopted i) the *number of residues* function, ii) the *number of cysteines* function, iii) the *labels global histogram* function, iv) the *labels local histogram* function and, v) the *labels local window* function. In addition to them, we defined three other feature functions directly computed from the primary sequence and four annotation-related feature functions. We now describe in detail all these feature functions. However, since only few of these features will effectively be selected, the reader can understand the rest of our study without considering the detailed descriptions of all candidate feature functions.

- *Number of residues*: returns the number of residues in the primary sequence.
- *Number of cysteines*: returns the number of cysteine residues in the primary sequence. This feature is made from the intuition that larger the number of cysteines is, larger the number of disulfide bonds will be, which usually lead to more stable structures.
- *Unnormalized global histogram*: computes twenty features, one per standard amino acid type, which are the numbers of residues of each type in the primary structure.
- *Position of residue*: returns the position 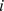 of the residue in the primary structure.
- *Relative position of residue*: computes one feature which is the residue position 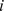 divided by the protein length 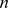. Although this feature may seem redundant with the previous one, the encoded information is different. The previous feature aims at encoding the absolute position of the residue with respect to the N-terminus. The intuition behind this feature is that the position of a residue might determine its disordered state (*e.g.*, the first four residues are prone to be disordered). Whereas, the relative position, which varies in 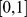, suggests a position regardless of the protein length.

We use the following notations to describe the annotation-related feature functions. For each type of annotation 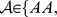 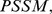 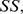 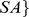, 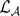 is the set of labels corresponding to 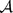 and 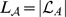 is the size of this set. We thus have: 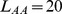, 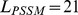 (the twenty amino acids and the gap), 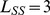, 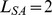. For a given primary structure of length 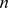, an annotation 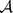 is represented as a set of probabilities 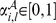 where 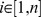 denotes the residue position and 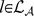 is a label. *E.g.*, 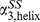 is the probability that the third residue of the protein is part of a helix.

In the general case, the 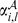 probabilities may take any value in range 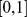 to reflect uncertainty about annotations. However, since the predictions made by SSpro and ACCpro are classes and that primary structures (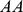) are always known perfectly, we have:
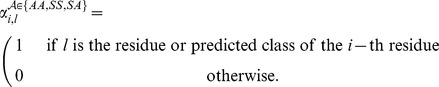



As PSSM elements typically range in 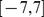, we scale them to 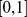 by using the function proposed in [30] and defined as following: 
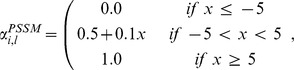
where 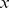 is the value from the raw profile matrix.

**Figure 4:**
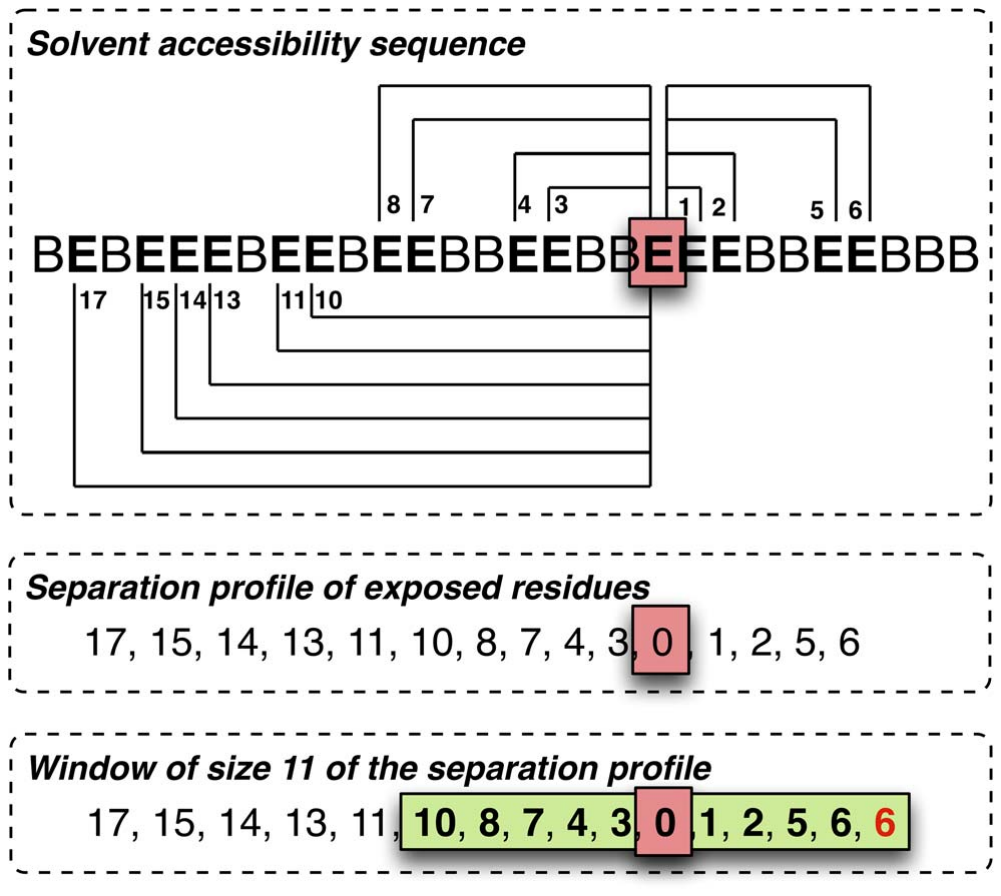
Illustration of the *separation profile window* function on exposed residues. Top: the functions first computes the amino acid distances that separate the residue of interest (highlighted by a red square). Middle: the separation profile of exposed residues. Bottom: the feature function returns the window (highlighted by a green rectangle) of size 11 centered around the residue of interest. In this example, the window slightly goes beyond the end of the sequence. As explained in the main text, in such cases we replace non available features by the maximal possible value, which is the 6 shown in red here.

For each annotation 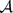, we have defined seven different feature functions:

- *Labels global histogram*: computes one feature per label 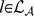, equal to 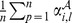.
- *Labels local histogram*: computes one feature per label 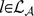 equal to 
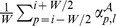
 and one special feature equal to the percentage of out-of-bounds positions, *i.e.*, positions 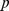 such that 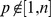.
- *Labels local window*: computes one feature per label 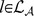 and per relative position 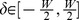, equal to 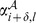. When the position is out-of-bounds, *i.e.*, 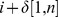, the feature is set to 0.
- *Separation profile window*: this feature function is inspired from the *cysteine separation profile window* function, which focuses on the distances that separate consecutive cysteine residues and encodes the distances around the cysteine residue of interest into features. According to the results presented in our previous work, this feature function led to an impressive improvement of our disulfide connectivity pattern predictor. Here, we propose a generalization of this function in order to be able to tackle any kind of annotation 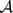. Figure 4 shows an illustration of a separation profile window of size 11 over exposed residues.

Given a residue position 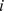, our generalized feature function describes the proximity of the 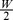 closest residues of the N-terminus side to the 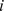-th residue (respectively, the 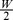 closest residues of the T-terminus side) that share a common label 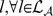. The proximity of a residue at the 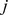-th position is expressed as the distance, in terms of number of amino acids in the primary structure, that separates the 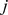-th from the 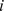-th residue, *i.e.*, 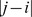. Note that, when using probabilistic predictors, the label of a residue is determined as the one with the highest probability 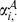.

When the number of residues that share 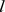 at the N-terminus side (respectively, at the T-terminus side) is insufficient, the missing distances are set to the greatest distance, *i.e*, the distance with the farthest residue that share 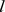 within the same terminus side.

- *Labeled segments window*: this is similar to the *labels local window* function except that rather than describing neighboring residues at position 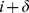, it describes neighboring segments 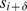. A segment consists in a sub-sequence of consecutive residues that share a common label 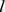, in the sense of the highest probability 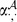.

Therefore, given a segment 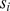, the function returns one description of this segment (in the form of feature vectors) per relative position 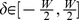. A segment 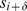 is described by 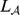 (one per label 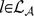) plus one features. Among the first 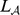 features, the one corresponding to the label of 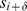 is equal to 1 while the other ones are set to 0. The last feature is the length of 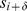. When the position 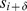 is out-of-bounds the features are all set to 0.

- *Dimeric global histogram*: this feature function is an extension of *labels global histogram* with the difference that instead of calculating the frequency of occurrence of each single label, it computes the frequency of occurrence of each pairs of labels. A pair of labels is formed by the labels of two consecutive residues (a word of size 2). The hope is that the distribution of some pairs of labels are significantly different in the case of disordered residues with respect to ordered ones. For example, a larger proportion of consecutive exposed residues may intuitively involve a larger disposition to form disordered regions. More formally, it returns one feature per pair of labels 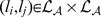, equal to 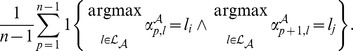

- *Dimeric local histogram*: this feature function is identical to the dimeric global histogram one except that it computes the frequency within a sliding window. More formally, given a residue position 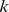, it returns one feature per pair of labels 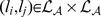 equal to 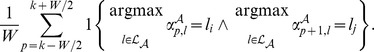

Our candidate feature functions are summarized in Table 2. Note that five of them are parameterized by window size parameters. To apply the feature function selection algorithm, we consider the following discrete sets of window sizes:

**Table 2:** Feature functions used in our experiments to encode residues.

Symbol	Parameter	d	Description
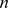	-	1	Number of residues
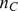	-	1	Number of cysteines
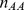	-	20	Unnormalized global histogram
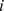	-	1	Position of residue
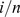	-	1	Relative position of residue
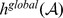	-	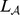	Labels global histogram
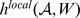	window size	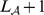	Labels local histogram
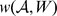	window size	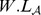	Labels local window
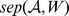	window size	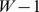	Separation profile window
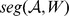	window size	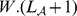	Labeled segments window
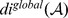	-	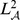	Dimeric global histogram
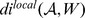	window size	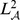	Dimeric local histogram

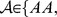
 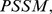 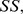 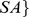. Symbols, parameters, number of features (d) and description of our candidate feature functions. Top: feature functions that are directly computed from the primary structure. Bottom: feature functions defined for every kind of annotation

- Local windows, separation profile window, labeled segments window and dimeric local histogram: 1, 5, 11, 15, 21.
- Local histograms: 10, 20, 30, 40, 50, 60, 70, 80, 90.

This setting leads to a total of 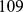 candidate features functions.

### Ensembles of extremely randomized trees

This tree-based ensemble method, proposed by Geurts *et al.* [31], is similar to the popular Random Forests approach [32]. The main differences with the latter are that extremely randomized tree ensembles (ETs) do not rely on bootstrap replicates (unlike the Random Forests method, each tree is built using all learning samples), and that cut-points are selected in a random fashion, which was shown to lead to better generalization performances [31]. The method has three hyper-parameters: 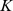, the number of random splits tested per node creation, 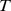, the number of trees composing the ensemble, and 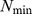, the minimum number of samples required to allow for splitting a node.

We use the probabilistic version of ETs, in which each leaf is associated with a probability of disorder, which is the empirical proportion of disordered residues among the training samples associated to that leaf. In order to make one prediction, we traverse each of the 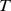 trees and return the average of the probabilities of disorder associated to the corresponding 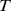 leaves.

## Results

This section describes our experimental study on disordered regions prediction. The first part presents the results of the main contribution of this paper, which aims at determining a relevant representation on Disorder723. The second part aim at constructing a model based on this relevant representation and ETs, and assessing this model on Casp10 and Pdb30. In the third part, we investigate the novel feature function and attempt to interpret its role in the prediction of disordered regions.

### Identification of a set of relevant feature functions

We now apply the feature function selection approach on top of ETs with the candidate feature functions of Table 2. We use a default setting of hyper-parameters of ETs that corresponds to an ensemble of 1 000 fully developed trees (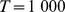, 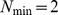) and 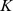 is set to the square root of the total number of features 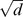, as proposed by Geurts *et al* [31].

To avoid any risk of over-estimation, we performed the selection on 10 different train/test splits of Disorder723. The performance measure being maximized by each run is the cross-validated AUC score of the training set. Table 3 reports the selected feature functions for each of the 10 independent runs. For the five iterations we consider, we observe that the selected feature functions on each of the 10 train/test splits are always 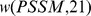, 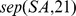, 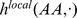 with a window size varying in 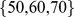, 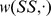 with a sliding window size in 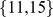 and 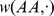 with a window size in 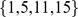. Regardless to window size parameters, the fact that we observed these feature functions during each run is very strong, since the selection algorithm has to select between 109 different candidate feature functions.

**Table 3:** Forward feature functions selection with 10 train/test splits.

Fold	Iteration 1	Iteration 2	Iteration 3	Iteration 4	Iteration 5
1	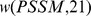	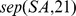	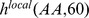	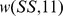	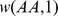
2	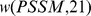	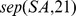	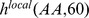	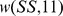	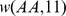
3	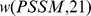	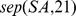	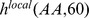	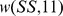	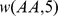
4	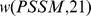	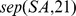	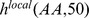	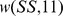	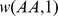
5	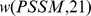	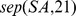	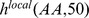	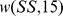	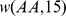
6	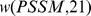	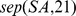	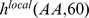	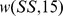	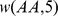
7	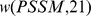	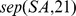	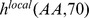	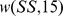	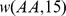
8	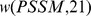	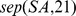	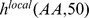	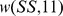	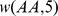
9	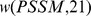	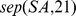	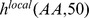	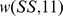	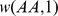
10	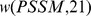	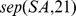	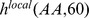	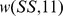	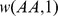
Mean					
Cross-validated	0.852 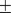 0.003	0.876 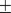 0.003	0.884 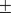 0.003	0.890 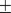 0.003	0.894 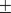 0.003
Validation	0.850 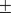 0.029	0.874 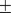 0.021	0.883 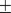 0.022	0.888 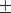 0.022	0.892 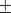 0.22

*Mean*: averages over the ten *cross-validated scores* and the ten *validation scores*. The cross-validated score is the mean of AUC scores obtained when cross-validating the training set of a run. The validation score is the AUC score obtained when evaluating the test set.

Note that, among the selected feature functions, two of them (the second and the fourth) rely on predicted structural annotations (the predicted solvent accessibility and the predicted secondary structure, respectively), which tend to show that predicted structural annotations contribute to make better disordered regions predictors.

Not surprisingly, the most important feature function detected by the selection is a sliding window of evolutionary information, which confirms that disordered regions differ from ordered regions in terms of their conservation profile. This feature function is also important for many other protein structure prediction tasks (*e.g.*, [1]).

On the other hand, the second most important feature function highlighted by our algorithm, namely 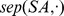, has - to our best knowledge - never been proposed in previous studies. Its discovery at a very early iteration was unexpected. It suggests that the proximities of a residue 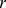 (in terms of amino acid positions in the primary sequence) to its nearest exposed or to its nearest buried residues are correlated with the fact that 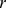 belongs to a disordered region. It is important to note the difference with 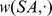. Indeed, 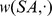 describes the solvent accessibility label of the flanking residues of 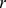. The proximity is fixed and limited by the number of flanking residues to take into consideration. Whereas, 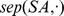 describes the inverse. Namely, it describes the proximity of the nearest residues to 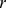 that correspond to fixed labels.

**Figure 5:**
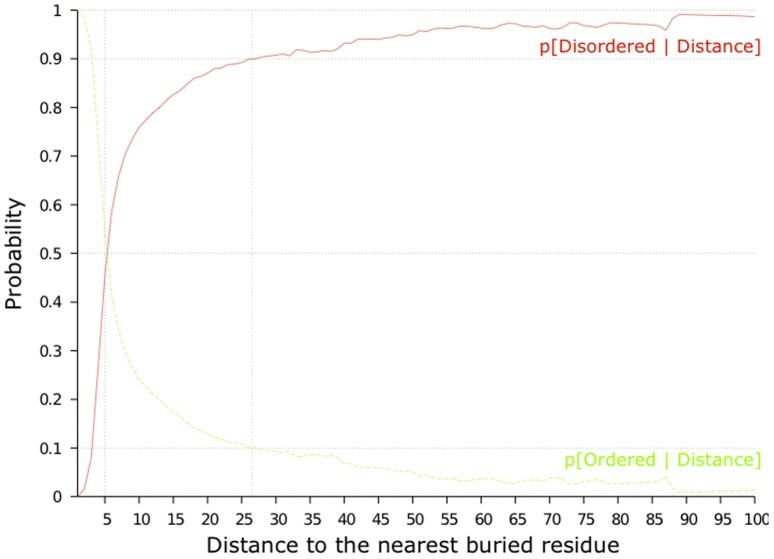
Probability of being (dis)ordered w.r.t. the distance to the nearest buried residue. For a given distance 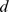, the probability 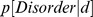 of being disordered is calculated as the portion of disordered residues among the residues that have their nearest buried residue located at a distance 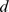. We computed these curves on the actual values of the solvent accessibility of Pdb30.

One way to explain the usefulness of this feature function is to look at the distributions of the distances that separate disordered (resp. ordered) residues to their nearest buried residue. Figure 5 shows the probability of a residue of being disordered (resp. ordered) according to the distance to its nearest buried residue, over the pdb30 dataset. We remark that the probability of a residue being disordered increases quickly when its distance to the next buried residue increases, and is above 0.5 as soon as the closest buried residue is at least 5 residues away.

Another important aspect of this discovery is that the 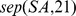 feature function is systematically detected just before the local amino acid composition 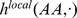 and far before 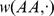. Indeed, these other two feature functions describe in different ways the sequence complexity, which is well-known to be low within disordered regions [5]. This therefore reinforces the fact that 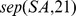 may be a key-aspect in our understanding of protein disordered regions and, consequently, protein structure-function relationships.

The fourth selected feature function is a short sliding window over predicted secondary structures 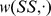. The usefulness of these features may be related to the strong difference between the distributions of predicted secondary structures within disordered regions with respect to ordered ones. For example, Table 4 shows that 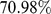 of disordered residues are predicted as coils against 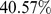 as it is the case with ordered residues and that solely 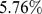 are predicted as sheets against 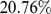 for ordered regions.

**Table 4:** Distribution of predicted secondary structure.

	Ordered		Disordered		Total	
Predicted helices	77,989	38.67%	3,235	23.26%	81,224	37.67%
Predicted sheets	41,874	20.76%	801	5.76%	42,675	19.79%
Predicted coils	81,840	40.57%	9,873	70.98%	91,713	42.54%
Total	201,703		13,909		215,612	

Distribution of the number of ordered/disordered residues and the total number of residues for each secondary structure class on Disorder723.

According to these results, we focus in the following on assessing the relevance of the feature functions 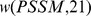, 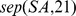, 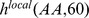, 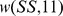 and 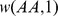, where we chose windows sizes by taking the most frequent sizes reported in Table 3. Indeed, contrarily to the observation made in [1] that suggested a very small number of relevant feature functions in the context of disulfide bridge prediction, the selection algorithm identified here a larger set of interesting feature functions.

### Evaluation of the selected feature functions

We now compare our models in terms of accuracy against a number of state-of-the-art methods on Disorder723, C10 and Pdb30. As previously, we use ETs with a default setting of its hyper-parameters. For each run, we use 80% of the training set to build an ensemble of trees predicting the probability to belong to a disordered region for a residue, and the remaining 20% to fix an ‘optimal’ decision threshold on this probability.

For Disorder723, we consider two baselines. Both evaluated their predictive performance using a 10-fold cross-validation on Disorder723. The first baseline is Cheng *et al.* [19], the authors of the Disorder723 dataset. They proposed an ensemble of 1D-recursive neural networks that reached an area under the ROC curve of 0.878. The second baseline is Eickholt *et al.* [33], who used boosted ensembles of deep networks to make predictions. They obtained a very high balanced accuracy (82.2%) and AUC (0.899).

The top of Table 5 reports our predictive performances when including successively the feature functions 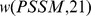, 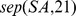, 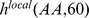, 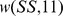 and 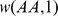, while the bottom of the Table 5 reports the scores of the two baselines from the literature. We observe that using only 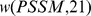 leads to a balanced accuracy (Acc) of 77.5%, an AUC of 0.853 and a F-measure of 49.6, which already outperforms the state of the art (46.3).

**Table 5:** Accuracy evaluation on the DISORDER723 dataset.

Features	Balanced Acc	Sensitivity	Specificity	AUC	F-measure
**10-fold cross validation of our algorithm over DISORDER723**					
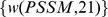					
	77.5 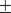 2.43	74.1 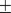 5.95	80.8 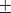 3.13	0.853 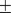 0.028	49.6 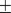 3.38
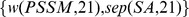					
	79.0 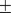 1.95	76.5 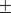 4.14	81.6 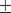 2.59	0.875 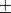 0.019	51.7 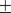 4.20
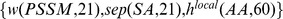					
	80.3 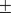 2.17	78.2 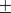 4.90	82.4 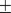 2.47	0.884 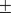 0.019	52.7 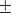 3.85
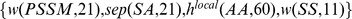					
	80.6 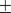 1.69	79.0 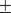 4.64	82.2 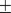 2.11	0.891 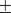 0.020	53.4 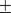 3.55
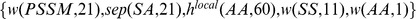					
	81.1 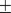 1.83	78.6 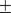 4.69	83.5 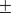 2.08	0.894 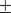 0.021	55.3 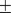 3.27
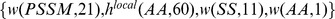					
	80.4 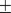 1.94	76.8 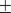 5.30	83.9 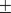 2.37	0.883 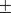 0.026	54.5 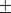 2.70
**Baselines tested on DISORDER723**					
Cheng *et al.* (2005) [19]	-	-	-	0.878	-
Eickholt *et al.* (2013) [33]	82.21 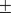 0.49	74.60 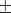 1.1	89.84 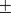 0.18	0.899 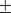 0.002	46.34 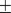 4.5

Top: the mean and standard deviation of the scores obtained when 10-folds cross-validating Disorder723 through the relevant feature functions. Bottom: baselines using Disorder723 to assess their model.

Moreover, we remark that by incrementally adding the remaining selected feature functions to the set systematically leads to significant improvements on Acc, AUC and F-measure. We have used the paired 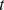-test on the AUC scores to statistically assess the significance of each increment. We noted that the corresponding 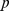-values (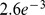, 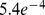, 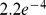 and 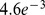) are well below the classical null hypothesis threshold (0.05). This observation reinforces the fact that the selected feature functions are relevant. When comparing our model based on all five selected feature functions to the state-of-the-art, we obtain a disordered regions predictor, which is very competitive in term of Acc (81.1%), equivalent in term of AUC (0.894) and clearly better in term of F-measure of 55.3. The middle of Table 5 shows the impact on the predictive performance of our model when we do not consider 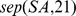 among the input feature functions. As expected, the scores significantly deteriorate with a 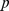-value of 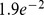 with respect to the model that comprise 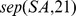. This observation reinforces the fact that this kind of feature function should be taken into account when predicting disordered regions.

To assess our models on Casp10, we compare our results against several baselines such as DNdisorder and PreDNdisroder, which were developed by Eickholt *et al.* [33]. Among the baselines, a number of them participated in the 10th CASP experiment. In order to make the comparison in a fair way, we construct our models on Disorder723 using feature functions that were selected according to Disorder723. Moreover, since Disorder723 does not contain any overlapping sequences with Caps10 and that Disorder723 was formed well before Casp10, we are in the same blind prediction setting than the participants of the competition.

The top part of Table 6 reports our results with the different sets of relevant feature functions while the bottom part of Table 6 reports the scores obtained by the baselines considered in [33]. Once again, we observe that enlarging the feature functions set systematically leads to significant improvements except for 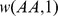. Two reasons may explain this phenomena, either the Casp10 dataset is too small and, consequently, prone to larger variances than big datasets, or the fifth iteration of the selection procedure starts to overfit Disorder723, which means that 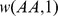 is not portable to other datasets. We believe that the second reason is more likely to be the true explanation, because the function 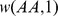 consists in discriminating disordered residues from ordered ones based on their amino acid type, which may be too dataset specific. As mentioned, the 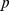-value of 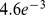 determined when including this feature set was indeed quite larger than those resulting from the inclusion of the other feature sets.

**Table 6:** Accuracy evaluation on the CASP10 dataset.

Features	Balanced Acc	Sensitivity	Specificity	AUC	F-measure
**Models learnt on DISORDER723 by our algorithm and tested on CASP10**					
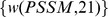					
	71.94 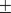 0.71	70.71 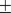 1.3	73.16 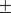 0.32	0.795 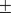 0.007	39.47 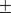 0.73
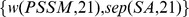					
	74.95 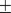 0.69	70.31 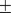 1.4	79.59 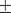 0.29	0.834 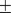 0.006	38.51 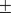 0.81
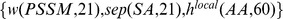					
	77.17 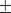 0.67	71.64 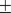 1.3	82.69 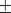 0.28	0.847 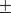 0.006	39.95 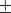 0.88
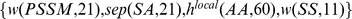					
	77.29 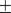 0.66	74.17 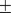 1.3	80.41 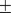 0.29	0.851 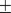 0.006	40.24 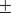 0.84
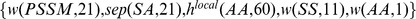					
	77.35 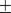 0.65	72.84 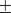 1.3	81.85 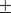 0.29	0.850 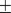 0.006	39.82 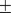 0.87
**Baseline performances on CASP10 as published by the CASP10 competition**					
metaprdos2 (340)	77.06 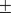 0.92	64.73 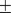 1.4	89.40 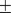 0.98	0.8727 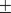 0.006	41.24 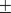 2.9
PreDisorder (125)	76.86 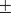 0.67	67.19 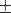 1.7	86.34 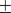 0.94	0.839 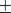 0.006	37.50 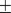 1.5
POODLE (216)	76.84 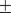 0.78	62.74 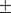 1.6	90.94 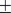 0.26	0.866 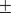 0.006	43.06 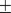 1.0
PreDNdisorder [6]	76.55 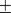 0.75	61.74 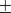 1.8	91.36 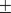 0.61	0.864 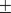 0.006	43.42 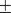 1.5
ZHOU-SPARKS-X (413)	75.68 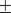 0.76	64.81 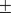 1.4	86.55 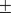 0.96	0.859 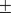 0.006	36.43 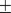 1.9
DNdisorder (424)	75.19 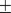 0.71	61.92 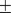 1.4	88.46 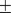 0.29	0.848 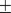 0.006	38.02 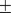 1.1
CSpritz (484)	75.13 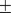 1.4	66.31 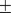 1.3	83.94 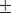 2.4	0.822 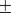 0.007	33.64 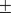 3.7
Espritz (380)	73.16 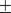 1.6	59.24 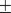 1.4	87.08 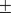 2.6	0.846 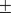 0.006	34.58 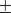 4.7
espritz_nopsi_X	71.98 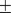 0.97	53.10 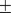 1.5	90.87 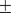 0.77	0.815 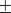 0.007	37.56 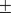 2.4
PrDOS-CNF (369)	70.35 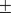 0.88	41.95 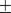 1.8	98.74 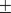 0.14	0.896 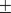 0.005	52.50 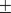 1.4
biomine_dr_mixed (478)	69.17 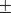 0.68	39.95 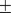 1.4	98.40 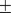 0.11	0.884 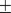 0.006	49.40 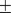 1.3
biomine_dr_pdb_c (228)	67.81 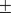 1.2	36.88 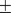 2.6	98.74 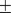 0.15	0.882 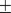 0.006	47.65 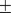 2.1
iupred_short	63.26 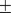 0.70	30.68 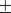 1.5	95.84 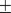 0.25	0.664 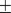 0.007	32.34 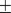 1.2

Top: the scores obtained when evaluating Casp10 on models learnt on Disorder723 through the relevant feature functions found on Disorder723. Bottom: comparison of a number of predictors, which participated in or evaluated their model to the 10th CASP experiment. These results were reported by [33]. In parenthesis: the group number of the methods that participated in the CASP10 experiment. The standard deviations were calculated by a bootstrapping procedure in which 80% of the dataset was sampled 1000 times, as it was done by [33].

According to Table 6, we remark that our model based on 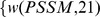, 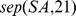, 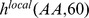, 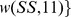 achieves excellent performances with respect to the state-of-the-art. We even slightly improve the state-of-the-art with a balanced accuracy of 77.29% against 77.06%, however, according to the variations, this improvement is not significant. We nevertheless outperformed the method of Eickholt *et al.* [33] (DNdisorder), which presented similar performances than our model on Disopred723.

**Figure 6:**
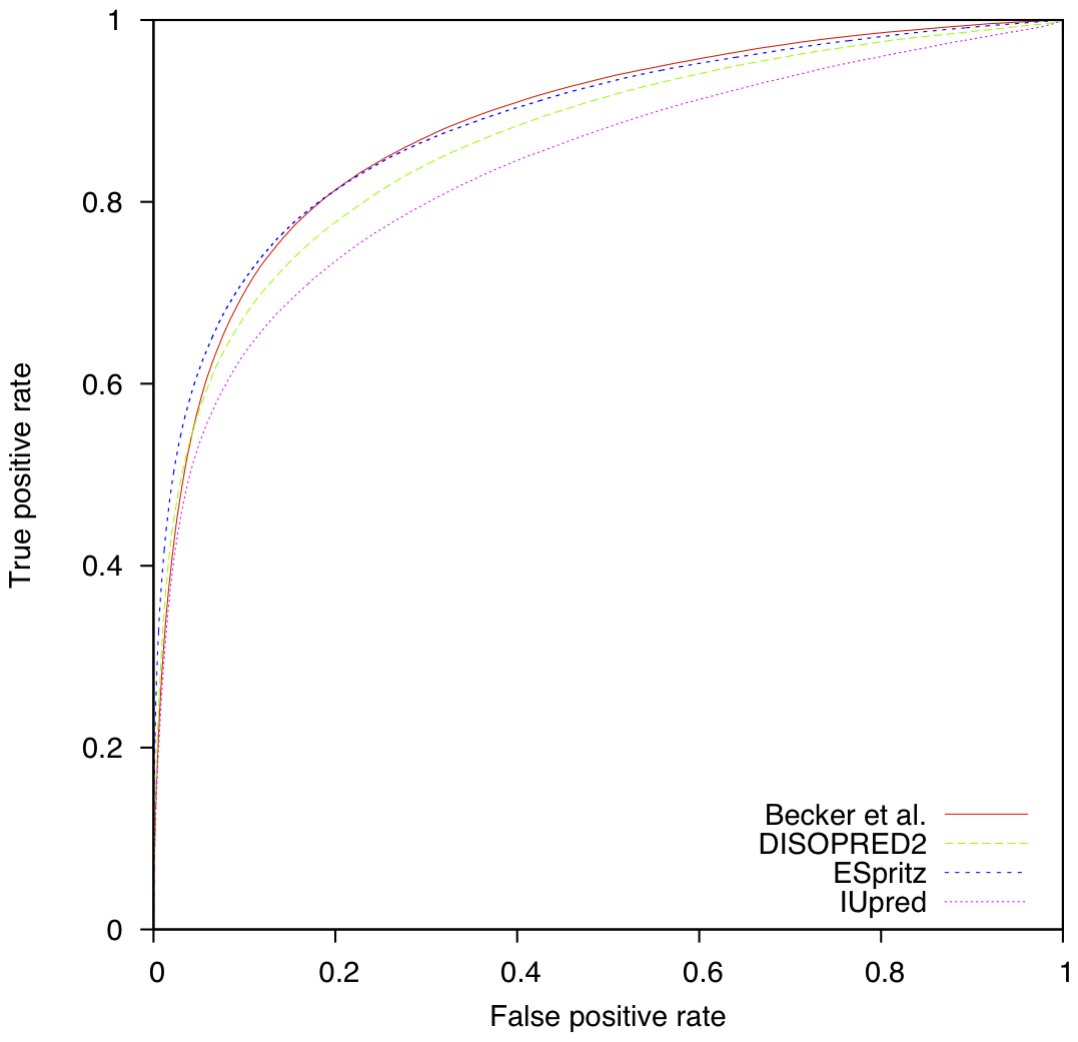
ROC curves on PDB30 dataset. ROC curve of our method (Becker *et al.*) and three freely downloadable predictors: DISPRED2 [34], ESpritz [36] and IUPred [35].

Although Casp10 is an entirely independent test set that had no detectable similarity to available structures at this time, its very limited size does not enable it to capture the universe of protein disorder. This is why we also evaluated our model on the far larger dataset Pdb30. Table 7 compares the predictive performances obtained by three freely and easily downloadable methods (DISOPRED2[34], IUPred[35] and ESpritz[36]) with respect to our model. We observe that our approach outperforms the three baselines with a balanced accuracy of 80.3% and presents a comparable area under the ROC curve (0.883) to ESpritz, even though our approach treats each residue independently, *i.e.*, without explicitly exploiting the key-fact that disordered regions are made of contiguous residues. Figure 6 shows the ROC curves for DISORDER2, ESpritz and IUpred on PDB30. We observe that our method and ESpritz are very close to each other and that ESpritz is slightly better in the low false positive rate.

**Table 7:** Evaluation on the PDB30 dataset.

Method	Balanced Acc	Sensitivity	Specificity	AUC	F-measure
Our method	80.36 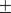 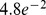	82.67 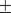 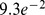	78.06 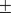 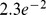	0.8835 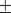 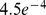	33.12 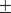 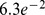
At 94.7% of specificity	76.73 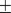 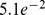	58.79 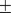 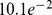	94.67 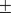 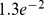	0.8835 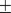 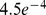	49.89 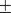 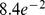
DISOPRED2 [34]	76.96 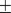 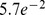	60.01 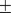 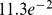	93.90 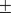 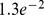	0.8658 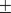 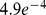	48.40 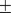 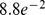
ESpritz [36]	78.49 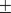 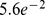	62.26 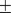 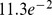	94.71 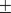 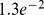	0.8856 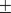 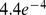	52.20 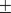 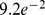
IUPred [35]	74.99 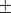 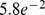	55.98 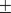 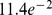	93.99 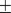 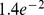	0.8363 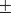 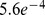	46.13 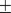 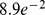

Predictive performances of three freely and easily downloadable methods on Pdb30. The standard deviations were calculated over the same 100 bootstrap copies of the whole dataset. Given the huge size of the dataset, all differences (even if they are sometimes tiny) are statistically significant. Notice that (except for the AUC calculation), our method uses a classification threshold that was selected on the training dataset (Disorder723) so as to maximize the balanced accuracy, which explains its difference in (sensitivity, specificity) pattern, as compared to the other methods. Changing the threshold so as to yield a 94.7% specificity on Pdb30, would reduce its sensitivity to 58.8%.

Note that since Pdb30 and Disorder723 are independent, the evaluation of our model is fair. However, we do not have access to the learning stage of the compared methods, which has possibly used sequences similar to the ones present in Pdb30. This may lead to an over-estimation of the predictive performance of those methods.

## Discussion

Predicting and understanding the nature of disordered regions is a key sub-problem of protein structure and function inference. This paper has adapted the algorithm presented in our previous work [1] on disulfide bridge prediction in order to identify the best way to represent protein residues in order to be usable by disordered region predictors. To this end, we used extremely randomized tree ensembles as an ‘off-the-shelf’ base learner in our feature function selection pipeline. We applied our approach to the Disorder723 dataset from the literature, so as to select relevant subsets of feature functions and to build simple residue-wise disorder prediction models.

Our experiments have shown that the combination of the feature functions 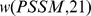 (a local window of size 21 of evolutionary information), 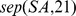 (a window of 21 of the separation profile of predicted solvent accessibility), 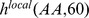 (a local histogram of size 60 of primary structure) and 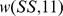 (a local window of size 11 of predicted secondary structure) is a relevant representation of protein residues in the context of disordered regions prediction.

From a biological point of view, the major contribution of this paper is the discovery of the 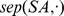 feature function, which has - to our best knowledge - never been highlighted as important in this context. This observation suggests that the proximities (in terms of amino acid distances) between consecutive exposed (and consecutive buried) residues should play a role in the formation of disordered regions and, consequently, in protein structure-function relationships.

To validate these observations with respect to the state-of-the-art in disorder prediction, we also evaluated our model on the set of proteins used in the Casp10 competition. On Casp10, our model constructed on the Disorder723 dataset turned out to obtain a very competitive assessment in terms of various predictive accuracy indicators, in spite of the fact that our work was focusing on feature identification rather than accuracy maximization. Since Casp10 is a small dataset that does not capture the whole universe of protein disorder, we further assessed our model on the independent and very large Pdb30 dataset, which contains 12,090 proteins and 2,991,008 residues. On Pdb30, our model obtained as well very competitive results with respect to three state-of-the-art methods, by clearly beating two of them and being at a tie with the third one.

From a methodological point of view, our paper also shows that the systematic feature family selection pipeline proposed in [1] and adapted here, is a viable and robust approach to yield interpretable information about relevant representations for protein structure inference and allows at the same time to build predictors with state-of-the-art accuracy. Still, it might be the case that extremely randomized tree ensembles with their defaults settings are not the best classifiers for disordered regions prediction. Also, in our predictors we treated each residue independently, *i.e.*, without taking advantage of the structured nature of the problem. Therefore, a main direction for future research is to evaluate more sophisticated classifiers using the feature functions highlighted by the present study.
